# Vascular features around the optic disc in familial exudative vitreoretinopathy: findings and their relationship to disease severity

**DOI:** 10.1186/s12886-023-02884-7

**Published:** 2023-04-05

**Authors:** Shuai Liu, Hongwei Zhao, Liuhui Huang, Cuixia Ma, Qiong Wang, Lei Liu

**Affiliations:** 1 Anhui Province Maternity and Child Health Hospital, Maternity and Child Health Hospital affiliated to Anhui Medical University, Hefei, 230001 China; 2 School of Information Science and Technology, University of Science and Technology of China, Hefei, 230022 Anhui China; 3 Department of Ophthalmology, Tenth People’s Hospital, Shanghai Tongji University School of Medicine, Shanghai, 200072 China

**Keywords:** Vascular features, Familial exudative vitreoretinopathy, Neonates, Computer technology, T-SNE visualization

## Abstract

**Background:**

Familial exudative vitreoretinopathy (FEVR) is a rare congenital disorder of retinal vascular development. We aimed to study the vascular characteristics around the optic disc in neonates with FEVR and the relationship with disease severity.

**Methods:**

A retrospective, case-control study including 43 (58 eyes) newborn patients with FEVR at stages 1 to 3 and 30 (53 eyes) age-matched normal full-term newborns was conducted. The peripapillary vessel tortuosity (VT), vessel width (VW) and vessel density (VD) were quantified by computer technology. The t-distributed stochastic neighbor embedding (t-SNE) algorithm was used to visualize the relationship between the severity of FEVR and the characteristics of perioptic disc vascular parameters.

**Results:**

The peripapillary VT, VW and VD were significantly increased in the FEVR group compared with the control group (*P* < 0.05). Subgroup analysis showed that VW and VD increased significantly with progressing FEVR stage (*P* < 0.05). And only VT in stage 3 FEVR was significantly increased compared with stage 1 and stage 2 (*P* < 0.05). After controlling the confounders, ordinal logistic regression analysis indicated that the VW (aOR: 1.75, *P* = 0.0002) and VD (aOR: 2.41, *P* = 0.0170) were significantly independent correlated with the FEVR stage, but VT (aOR: 1.07, *P* = 0.5454) was not correlated with FEVR staging. Visual analysis based on the t-SNE algorithm showed that peri-optic disc vascular parameters had a continuity along the direction of FEVR severity.

**Conclusions:**

In the neonatal population, there were significant differences in peripapillary vascular parameters between patients with FEVR and normal subjects. Quantitative measurement of vascular parameters around the optic disc can be used as one of the indicators to assess the severity of FEVR.

## Introduction

Familial exudative vitreoretinopathy (FEVR), first described by Criswick and Schepens in 1969, is a rare disorder of retinal vascular development that primarily affects retinal angiogenesis, leading to hypervascularization and poor vascular differentiation in the peripheral retina [1]. It is a highly genetically and phenotypically heterogeneous disease. The mode of inheritance can be expressed as autosomal dominant, autosomal recessive, and X-linked recessive inheritance [2–4]. Mild forms of FEVR are usually asymptomatic and show only peripheral vascular abnormalities such as peripheral avascular areas, vitreoretinal adhesions, arteriovenous anastomosis, redundant vascular branches, and V-shaped areas of chorioretinal degeneration [5, 6]. More severe FEVR can manifest as a combination of macular and vascular dragging, radial retinal folds, traction retinal detachment, preretinal vitreous tissue, vitreous hemorrhage, and subretinal exudation [6].

Peripheral retinal abnormalities have always been the focus of FEVR research. With the development of fundus imaging technology, more and more evidence show that retinal vascular development failure caused by FEVR not only affects the peripheral retina, but also affects the macula. Yonekawa et al. described various macular structural abnormalities including vitreomacular traction, diminished foveal contour and cystoid macular edema [7]. Chen et al. indicated FEVR status is associated with a significantly smaller foveal avascular zone, a thicker fovea, an abnormally preserved inner retinal layer in fovea, and decreased vessel density (VD) in both the superficial and deep layers of parafoveal area. [8]. Koulisis et al. used OCTA to study the changes in the macular microvascular and capillary network in FEVR patients, and revealed that early FEVR patients manifested decreased VD, reduced vascular branches and larger caliber vessels in the superficial and deep capillary plexus [9]. Zhang et al. revealed that there may be parafoveal microvascular defects in FEVR and the loss of VD in superficial capillary plexus may be associated with the severity of FEVR [10]. These previous findings suggest that patients with FEVR also have marked developmental defects in the macula and retinal capillary network.

To date, few studies have been reported on the characteristics of the optic disc and its surrounding vessels in patients with FEVR. Yuan et al. determined that patients with asymptomatic FEVR had more blood vessels radiating from the optic disc at the posterior pole, a significantly larger disc-to-macular distance as well as a remarkably smaller optic disc with a decreased horizontal diameter [5, 11, 12]. By analysis of color fundus photographs captured by RetCam, Kashani et al. reported severe venous dilation and tortuosity in the posterior pole of some FEVR patients. Arterial tortuosity is also observed around the optic disc in FEVR patients with significant vascular traction [6]. Based on the above findings combined with our clinical observations, the authors hypothesized that the peri-optic disc vasculature in patients with FEVR also differs from the normal population. In their study, Kashani et al. only qualitatively reported the tortuosity and dilation of the peripapillary vessels, and lacked further in-depth quantitative exploration of the changes in vascular characteristics [6]. In addition, the current research on FEVR mainly focuses on adults and children, and there are few reports on the clinical features of FEVR in neonates. Since the fundus screening of full-term infants was carried out in China, more and more neonatal patients with FEVR have been identified, which has laid the foundation for conducting studies in the neonatal population.

Therefore, this study aimed to quantitatively analyze the vascular characteristics around the optic disc, including vascular tortuosity, width and density, based on the fundus images of neonatal FEVR patients taken by a wide-field fundus imaging system (RetCam 3) with the aid of computer technology. At the same time, the relationship between the peri-optic disc vascular parameters and the severity of FEVR was further analyzed. The quantitative evaluation of vascular parameters in the posterior pole of FEVR patients can further reveal some disease characteristics that are difficult to detect with the naked eye, which is of great significance for a comprehensive insight into the pathogenesis of FEVR.

## Methods

This was a retrospective, case-control study. Patients were full-term newborns who underwent routine fundus screening within 7 days of birth at Anhui Province Maternity and Child Health Hospital between September 01, 2017 and May 31, 2021. A total of 43 (58 eyes) FEVR patients from stage 1 to 3 were included in this study, and 30 (53 eyes) age-matched normal full-term neonates were included as controls. All participants underwent fundus examination using a wide-field fundus imaging system RetCam 3 (Clarity Medical Systems, Inc, Pleasanton, CA, USA), and patients suspected of FEVR were included after confirmation of diagnosis by genetic testing (high-throughput next-generation sequencing technology). The study adhered to the principles of the Declaration of Helsinki and was approved by Ethics Committee of the Anhui Province Maternity and Child Health Hospital of Anhui Medical University.

**Fig. 1:**
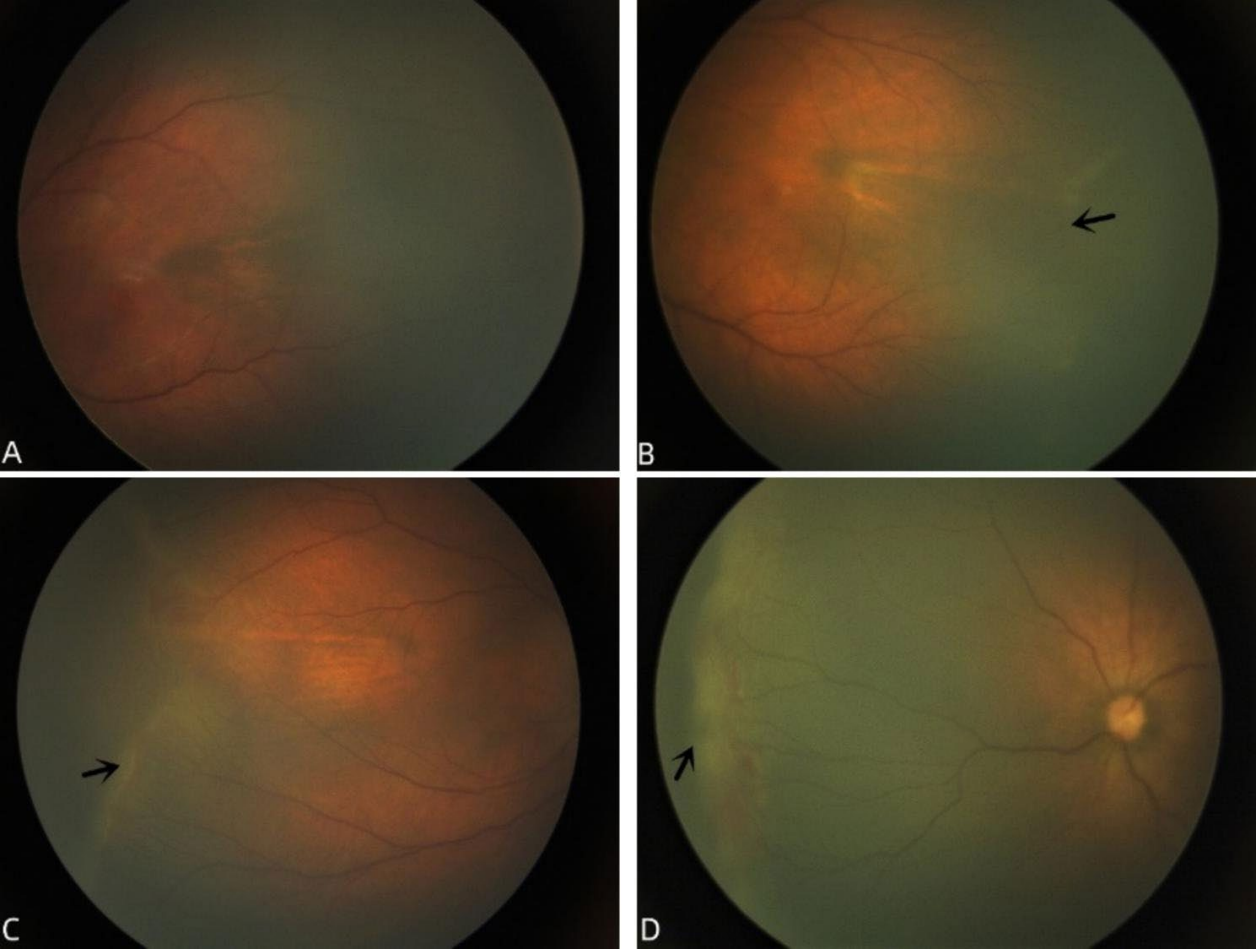
(A-D). Typical Fundus Images of Normal Full-term Newborns and Different Stages of FEVR Patients Taken by RetCam 3.A: Fundus image of a normal full-term neonate, retinal blood vessels developed to the peripheral retina; B: Fundus images of FEVR patients at stage 1, the demarcation line of avascular zone can be seen in the temporal periphery of the retina. C: Fundus image of FEVR patients at stage 2, ridge-like changes can be seen in the temporal periphery of the retina with neovascular tissue surrounding the ridge. D: Fundus image of FEVR patients at stage 3, ridge-like changes were seen in the nasal periphery of the retina with hemorrhage and fibrous tissue proliferation, causing peripheral retinal detachment without involving the macula

To ensure the reliability of the diagnosis of FEVR patients, we adopted strict inclusion criteria as follows: (1) the presence of avascular areas in the peripheral retina of both eyes as confirmed by a wide field fundus imaging system (RetCam 3); (2) genetic test results indicating the presence of at least one pathogenic mutation in 5 genes, including *FZD4, LRP5, NDP, TSPN12 and ZNF408*; (3) newborns who underwent fundus examination within 7 days of birth; (4) normal full-term newborns with no history of oxygenation. Exclusion criteria: (1) FEVR patients with retinal detachment involving the macula or total retinal detachment; (2) FEVR patients with retinal folds or vitreous hemorrhage; (3) patients with non-FEVR vitreoretinal pathology (Norrie disease, persistent fetal vascular system, or retinopathy of prematurity) were also excluded. Patients were staged based on the FEVR clinical staging system suggested by Pendergast [13]: stage 1, peripheral avascular area; stage 2, retinal neovascularization; stage 3, extramacular retinal detachment; stage 4, retinal detachment involving the macula; stage 5, complete retinal detachment. All FEVR patients were staged according to the highest stage of disease in each eye by two experienced pediatric ophthalmologists (Shuai Liu and Liuhui Huang) based on RetCam 3 fundus images (see Fig. 1).

**Fig. 2:**
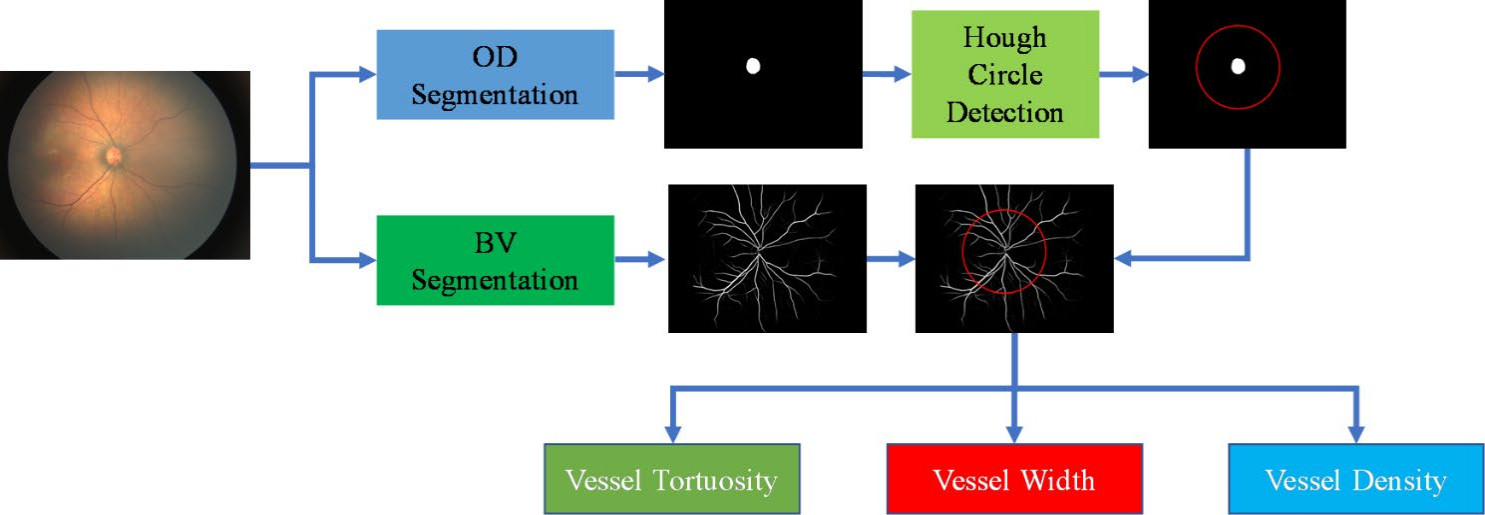
The pipeline of FEVR analysis

The fundus images of the subjects were acquired using a wide-field fundus imaging system (RetCam 3), and then the characteristics of blood vessels (BV) around the optic disc (OD) were analyzed by computer technology. All fundus images in this experiment meet screening criteria as follows: (1) visible optic disc, blood vessels and macula; (2) clear quality standards for assessing the disease. Figure 2 depicts the overall flow of performing FEVR analysis. First, the OD and BV were segmented using the U-net segmentation network to obtain optic disc and blood vessel segmentation results. Then, the Canny edge detection method was performed on the segmented optic disc image, and the center and radius of the optic disc (OD*r*) were obtained using the Hough circle detection algorithm. We delineated the region of interest (ROI) with the center of the OD, as shown by the red circle in Fig. 2. Finally, the ROI was applied to the segmented vessel image, and the vessel tortuosity (VT), vessel width (VW), and vessel density (VD) in the ROI were calculated.

In this study, there are two U-Net segmentation models: BV segmentation and OD segmentation [14]. The code adopts open source project development on GitHub (https://github.com/orobix/retina-unet). For BV segmentation, all fundus images in this study were resized to a common resolution of 640 × 480 pixels and the U-Net was trained on 500,000 image patches (48 × 48 pixels) extracted from 200 retinal images, with manually labeled images of blood vessels used as ground truth. The manual segmentation method has been published in a previous paper [15]. The trained U-Net was used to segment the pictures completely by removing overlapping patches and averaging the model predictions to obtain a “vesselness” probability map. The trained U-Net was used to perform a complete segmentation of the image by extracting overlapping patches and averaging the model predictions to generate a “vessel” image. For OD segmentation, ORIGA-650 [16] was employed to train the OD U-Net segmentation network (the same as vessel segmentation), which contains 650 fundus images from 168 glaucomatous eyes and 482 normal eyes. The manual ground truth of the optic disc and cup mask was included. All of the optic disc and optic cup masks were manually annotated by ophthalmologists.

According to the study [17], the typical diameter of the human OD is 1.83 mm, and the distance from the center of the fovea to the center of the OD is 4.93 mm, which is about 5 times the OD radius (ODr). To cover the entire the macula and the OD, in line with the study [18], we adopt a circular area with a radius of 6×ODr as the ROI. The ROI encompasses the macula and the OD, allowing us to concentrate on the important vessels.

**Fig. 3:**
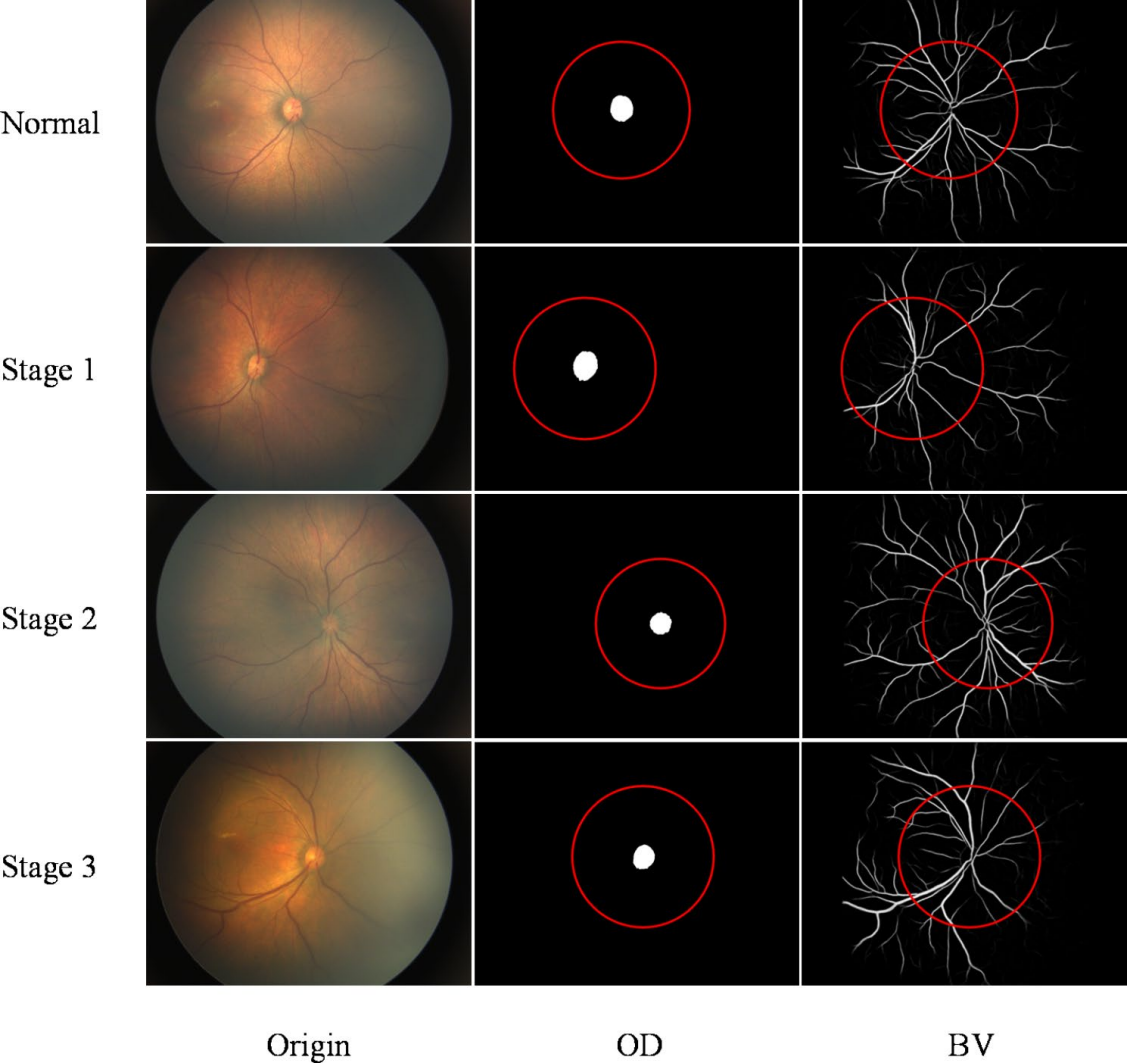
Original images, segmented OD and BV for the normal, Stage 1, Stage 2 and Stage 3 of FEVR conditions. The red circles, with a radius of $6\times {OD}_{r}$, indicate the ROI.

In Fig. 3, the first column represents the original image (Origin), and the second and third columns represent the optic disc map and vessel map obtained by the Artificial Intelligence segmentation model, respectively. The first row, the second row, the third row, and the fourth row represent the original images and segmentation result images of Normal, FEVR 1, FEVR 2, and FEVR 3 images, respectively. Specially, the quantitative analysis method analyzes only the VT, VW and VD within the ROI.

Based on the results of vessel segmentation in retinal images, we examined the morphological characteristics of the vessels surrounding the OD, as shown in Fig. 1(B). As a metric of VT, we used arc-length normalized total squared curvature [19]. To compute VT, (1) we first extracted the number of blood vessels; Then (2) we calculated the tortuosity of each blood vessel; Finally (3) we took the average of all VT as the tortuosity of the entire image. The formula for determining VT is as follows:

1 $$\varvec{T}\varvec{o}\varvec{r}\varvec{t}\varvec{u}\varvec{o}\varvec{s}\varvec{i}\varvec{t}\varvec{y}={\int }_{{\varvec{t}}_{0}}^{{\varvec{t}}_{1}}{\varvec{k}\left(\varvec{t}\right)}^{2}\varvec{d}\varvec{t}$$

where $\varvec{k}\left(\varvec{t}\right)$ represents the curvature.

To obtain the average width of vessels within the ROI region, we utilized the Euclidean distance transform [20] to evaluate the distance of each pixel of the vessel to the nearest background pixel. We extracted the centerline of the vessel and calculate the width of a single vessel by applying an adaptive threshold method [21]. The mean VW was calculated as follows:

2 $$\varvec{W}\varvec{i}\varvec{d}\varvec{t}\varvec{h}=\frac{1}{\varvec{N}}\sum _{\varvec{i}}^{\varvec{N}}{\varvec{W}}_{\varvec{i}}$$

where ${\varvec{W}}_{\varvec{i}}$ represents the width of i-th vessel in ROI.

Following the paper [22], retinal VD was evaluated using a computer-aided image analysis tool by automatically segmenting all visible retinal vessels and calculating the area of vessels in several circles around the optic disc. VD was calculated as the ratio of vessel area to ROI area without the OD. The following is the calculating formula:

3 $$\varvec{D}\varvec{e}\varvec{n}\varvec{s}\varvec{i}\varvec{t}\varvec{y}=\frac{{\varvec{A}}_{\varvec{v}\varvec{e}\varvec{s}\varvec{s}\varvec{e}\varvec{l}}}{{\varvec{A}}_{\varvec{i}\varvec{m}\varvec{a}\varvec{g}\varvec{e}}}$$

where ${\varvec{A}}_{\varvec{v}\varvec{e}\varvec{s}\varvec{s}\varvec{e}\varvec{l}}$ and ${\varvec{A}}_{\varvec{i}\varvec{m}\varvec{a}\varvec{g}\varvec{e}}$represent the vessel area and the ROI area without OD, respectively.

t-distributed stochastic neighbor embedding (t-SNE) is a statistical method that is used for reducing dimensionality and visualization [23]. The operation process of t-SNE is as follows: (1) To begin, a probability distribution is established over two high-dimensional objects, with a greater probability assigned to comparable items and a lower value assigned to dissimilar points. (2) Second, in the lower dimensional space, it generates an analogous probability distribution over the points, and the Kullback-Leibler divergence (KL divergence) between the two distributions is lowered with regard to the map’s point positions.

In this research, we employed the t-SNE method to visualize the vessel features (VT, VW and VD) proposed in this paper, revealing the potential relationship between these features and disease severity.

Statistical analysis of data was performed using SAS Statistics software version 8.02 (SAS Institute Inc, Cary, NC, USA). The measurement data were expressed as mean ± standard deviation. According to the normality test, comparisons of means between two groups were performed using the unpaired t-test or Wilcoxon-Mann-Whitney test. And comparisons among groups were performed using the Kruskal-Wallis test or one-way ANOVA. An ordinal logistic regression model was used to determine the correlation between perioptic disc vascular parameters and different stages of FEVR patients, with adjusted odds ratios (aOR) and 95% confidence intervals (CI) estimated to minimize confounding factors. Variables entered into the model included VT, VW, VD, sex, age, gestational age at birth, and birth weight. *P* values less than 0.05 were defined as statistically significant.

## Results

### Participant demographics

Table 1 shows the participant demographics. A total of 43 (58 eyes) neonatal FEVR patients with stage 1 to 3 and 30 (53 eyes) age-matched normal full-term neonates were included in this study. We excluded 28 eyes in the FEVR group because low quality of fundus images in peripheral area could not meet the criterion of diagnosing the stage of FEVR. And seven eyes in the control group were excluded for the same reason. The age of patients in the FEVR group was 1–6 days, with an average of 2.26 ± 1.29 days, and the age of the control group was 1–6 days, with an average of 2.67 ± 1.21 days. The gestational age of patients at birth in the FEVR group was 264–291 days, with an average of 275.00 ± 8.32 days, and the gestational age of the control group was 266–287 days, with an average of 275.97 ± 5.70 days. The birth weight of patients in the FEVR group was 2750-4030 g, with an average of 3266.51 ± 420.46 g, and that of the control group was 3000-3900 g, with an average of 3385.83 ± 263.87 g. Additional detailed demographic information is presented in Table 1.

**Table 1:** Participant Demographics

	Control	FEVR	*P* Value
No. of patients	30	43	—
No. of eyes	53	58	—
Sex (female vs. male)	11vs 19	13 vs. 30	0.5648#
Age (days), mean ± SD	2.67 ± 1.21	2.26 ± 1.29	0.1060##
Gestational age (days), mean ± SD	275.97 ± 5.70	275.00 ± 8.32	0.5578###
Birth weight (g), mean ± SD	3385.83 ± 263.87	3266.51 ± 420.46	0.2942##
No. of Mutant genotype (%) in Patients of FEVR			
*LRP5*	—	17(39)	—
*FZD4*	—	11(26)	—
*NDP*	—	7(16)	—
*TSPN12*	—	6(14)	—
*ZNF408*	—	2(5)	—
No. of eyes (%) With FEVR by stage			
Stage1	—	18(31)	—
Stage2	—	28(48)	—
Stage3	—	12(21)	—

# chi-square test.

## Wilcoxon-Mann-Whitney test.

###Unpaired t-test.

### Changes in mean peri-optic disc vascular tortuosity in FEVR patients compared with the control group

Based on the fundus images captured by RetCam 3, we first quantitatively analyze the vascular characteristics around the optic disc with the aid of computer technology. The results indicated that the mean vascular tortuosity (VT) around the optic disc in the FEVR group was significantly increased compared with the control group, 72.43 ± 3.58 (10^4^ cm^− 3^) and 70.80 ± 4.05 (10^4^ cm^− 3^) (*P* = 0.0266), respectively (see Table 2). Superficially, the VT increased with the progression of the FEVR stages, but further subgroup analysis showed that only the VT of the 3-stage FEVR was significantly higher than that of the control group(P = 0.0018), stage 1 (P = 0.0025) and stage 2 (P = 0.0361). There was no significant difference in VT between the other groups (P > 0.05) (see Table 3).

**Table 2:** Vessel Parameters between Control and FEVR Groups

	Control	FEVR	*P* Value
VT (10^4^ cm^− 3^)	70.80 ± 4.05	72.43 ± 3.58	**0.0266**
VW(µm)	63.46 ± 3.30	66.42 ± 3.52	**< 0.0001**
VD(%)	4.59 ± 0.69	5.29 ± 1.13	**0.0018 #**

Values in bold font are statistically significant at P < 0.05.

Data are mean ± standard deviation.

Statistics are Unpaired t-test unless other indicated.

*#* Wilcoxon-Mann-Whitney test.

VT = vessel tortuosity; VW = vessel width; VD = vessel density.

**Table 3:** Vessel Parameters between Control and Different Stages of FEVR Groups

		Stages of FEVR			*P* Value					
	Control	Stage1	Stage2	Stage3	Stage 1 vs. Control	Stage2 vs. Control	Stage3 vs. Control	Stage 1 vs. Stage2	Stage 1 vs. Stage3	Stage 2 vs. Stage3
VT (10^4^ cm^− 3^)	70.80 ± 4.05	71.09 ± 2.83	72.22 ± 3.65	74.91 ± 3.42	0.7766	0.1241	**0.0018**	0.2720	**0.0025**	**0.0361**
VW(µm)	63.46 ± 3.30	63.68 ± 2.32	66.33 ± 2.34	70.76 ± 3.10	0.7975	**0.0001**	**< 0.0001**	**0.0005**	**< 0.0001**	**< 0.0001**
VD(%)	4.59 ± 0.69	4.61 ± 0.85	5.21 ± 1.00	6.50 ± 0.79	0.6531#	**0.0079#**	**< 0.0001#**	**0.0290#**	**< 0.0001#**	**0.0004#**

Values in bold font are statistically significant at P < 0.05.

Data are mean ± standard deviation.

Statistics are Unpaired t-test unless other indicated.

# Wilcoxon-Mann-Whitney test.

VT = vessel tortuosity; VW = vessel width; VD = vessel density.

### The average vascular width around the optic disc in the FEVR patients was significantly increased compared with the control group

The average peripapillary vascular width (VW) in the FEVR patients was significantly increased compared with the control group. Quantitative analysis showed that the mean VW around the optic disc in the FEVR group was significantly increased compared with the control group, 66.42 ± 3.52(µm) and 63.46 ± 3.30 (µm), respectively (P < 0.0001, see Table 2). Further subgroup analysis revealed that with the progression of the FEVR stage, the VW increased significantly, and the differences in VW between the FEVR subgroups were statistically significant (stage 1 vs. stage 2, P = 0.0005); (stage 1 vs. stage 3, P < 0.0001); (stage 2 vs. stage 3, P < 0.0001). The VW in the 2-stage and 3-stage FEVR groups was significantly higher than that in the control group, and the VW in the 1-stage FEVR group was not significantly different from that in the control group (stage 1 vs. control, *P* = 0.7975); (stage 2 vs. control, *P* = 0.0001); (stage 3 vs. control, *P* < 0.0001) (see Table 3). The above results show that the caliber of blood vessels around the optic disc in FEVR patients was significantly larger than that in the control group, and with the progression of stages, the caliber of blood vessels continued to increase.

### The vascular density around the optic disc in the FEVR patients was significantly increased compared with the control group

The perioptic disc vascular density in the FEVR patients was significantly increased compared with the control group. The results indicated that the peripapillary vascular density (VD) in the FEVR group was significantly higher than that in the control group, 5.29 ± 1.13(%) and 4.59 ± 0.69(%), respectively (*P* = 0.0018) (see Table 2). Further subgroup analysis showed that with the progression of the FEVR stage, the VD increased significantly, and the differences in VD between the subgroups of FEVR were statistically significant (stage 1 vs. stage 2, *P* = 0.0290); (stage 1 vs. stage 3, *P* < 0.0001); (stage 2 vs. stage 3, *P* = 0.0004). The VD in the 2-stage and 3-stage FEVR groups was significantly higher than that in the control group, and the VD in the 1-stage FEVR group was not significantly different from the control group (stage 1 vs. control, *P* = 0.6531); (stage 2 vs. control, *P* = 0.0079); (stage 3 vs. control, *P* < 0.0001) (see Table 3). The above results suggest that with the progression of the FEVR stage, the VD around the optic disc increases continuously.

### The vascular width and blood vessel density around the optic disc in patients with FEVR are significantly correlated with disease staging

Table 4 shows the correlation between the perioptic disc and the FEVR staging. We used an ordinal logistic regression model to further explore the correlation between peripapillary vascular parameters and FEVR staging. The results showed that VW (aOR, 1.75, 95% CI:1.30–2.34, *P* = 0.0002), VD (aOR, 2.41, 95%CI:1.17–4.97, *P* = 0.0170) was significantly independent associated with FEVR staging, after adjusting for confounders such as sex, age, gestational age, and birth weight, and VT (aOR, 1.07, 95% CI: 0.87–1.32, *P* = 0.5454) was not correlated with FEVR staging (see Table 4). These results suggest that in patients with the FEVR stages 1 to 3, changes in peripapillary VW and VD may reflect disease severity (based on their stages).

**Table 4:** The Correlation of Vessel Parameters with The FEVR Staging

	Staging Groups of FEVR				Analysis #	
	Stage 1	Stage 2	Stage 3	*P* Value	aOR (95% CI)	*P* Value
VT (10^4^ cm^− 3^)	71.09 ± 2.83	72.22 ± 3.65	74.91 ± 3.42	**0.0129**##	1.07 (0.87–1.32)	0.5454
VW(µm)	63.68 ± 2.32	66.33 ± 2.34	70.76 ± 3.10	**< 0.0001**##	1.75 (1.30–2.34)	**0.0002**
VD(%**)**	4.61 ± 0.85	5.21 ± 1.00	6.50 ± 0.79	**< 0.0001###**	2.41 (1.17–4.97)	**0.0170**

Values in bold font are statistically significant at P < 0.05.

Data are mean ± standard deviation.

Statistics:

# Ordinal logistic regression; ## one-way ANOVA; ### Kruskal-Wallis test.

# Ordinal logistic regression model, adjusted by sex, age, gestational age and birth weight. The variables are as described in the table above.

VT = vessel tortuosity; VW = vessel width; VD = vessel density.

### Visualization of FEVR severity with peri-optic disc vascular characteristics

**Fig. 4:**
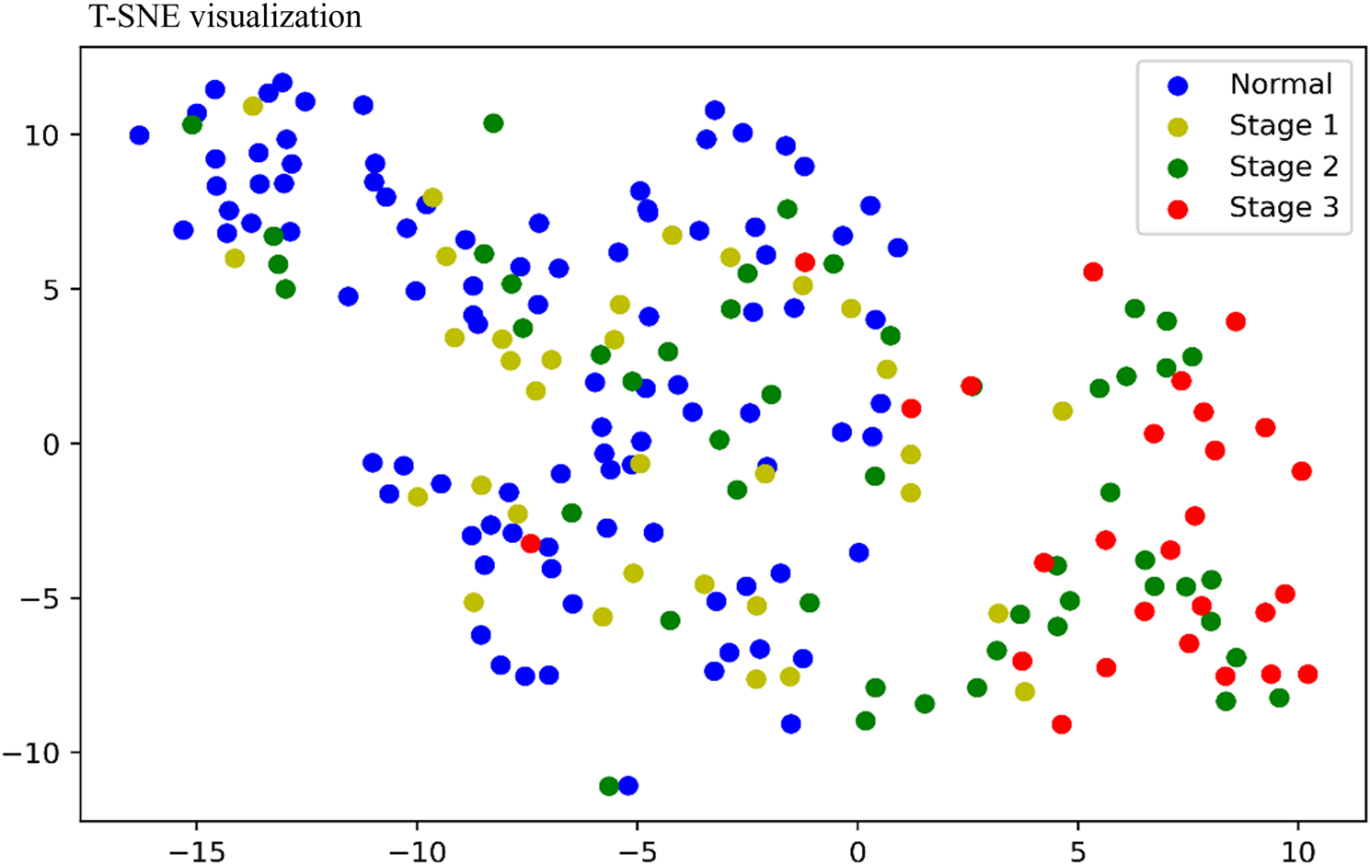
T-Distributed Stochastic Neighbor Embedding (T-SNE) visualization of BV features for analysis of FVER severity

Figure 4 shows the t-SNE algorithm used to visualize FEVR severity with the vascular features (VT, VW, VD) analyzed in this paper. Each point on the scatter plot corresponds to a sample, with blue indicating normal, yellow indicating Stage 1, grey indicating Stage 2, and red indicating Stage 3. From Fig. 4 we can find that similar samples (based on their characteristics) appear closer to each other than dissimilar ones. t-SNE visualizing demonstrates a qualitative separation between different disease grades. Normal partially overlaps with Stage 1 FEVR and Stage 2 FEVR, but barely overlaps with Stage 3 FEVR, where Stage 1 FEVR is intermediate between Normal and Stage 2 FEVR, and Stage 2 FEVR is intermediate between Stage 1 FEVR and Stage 3 FEVR, demonstrating continuity of disease severity. Overall, the visualization in Fig. 4 FEVR shows that the vascular features analyzed in this paper have a continuum along the direction of disease severity. The visualization revealed strong trends in illness characteristics of various severity levels, and the qualitative analysis lays the foundation for future completely automated FEVR disease severity assessment and treatment monitoring.

## Discussion

Since the fundus screening of full-term neonates was conducted in China, more and more neonatal patients with FEVR have been identified, and the incidence of FEVR in newborns has been reported to be about 1.19% [24]. Because FEVR has a tendency for life-long recurrence, for children under 3 years old, regular fundus examination using RetCam is the main way to monitor the progression of the disease. However, repeated fundus examinations often lead to low compliance for the families of children. Therefore, how effectively monitoring the changes in FEVR has become a nonnegligible clinical problem. Compared with the peripheral retinal images, the images of the posterior pole of the fundus are easier to obtain. Therefore, by studying the differences in peripapillary vascular features between FEVR patients and the normal population, we can not only understand the clinical characteristics of FEVR more comprehensively, but also may provide a new basis for further evaluation and monitoring of changes in FEVR.

We quantified the mean peripapillary VW and VT, and showed that FEVR patients had more dilated and tortuous perioptic disc vessels compared to the control group. This is consistent with the clinical observations of Kashani et al. [6]. Kashani et al. qualitatively revealed the tortuosity and dilation of the peripapillary vessels in the posterior pole of some FEVR patients based on the color fundus photographs captured by RetCam. Our findings also confirm the observations based on animal experiments. Xu et al. showed that major arteries and veins were present in the retinal vitreous surface of adult *Fzd4*^−/−^ mice, but they appeared enlarged and tortuous [25]. Chen et al. showed that at P12, the retina of *Lrp5*^−/−^ mice began to develop dilated blood vessels, with enlarged microaneurysm-like lesions in the superficial layer. And the progression of these lesions is seen at P17, persisting into adulthood [26].

Further subgroup analysis showed that the diameter of perioptic disc vessels in FEVR patients increased significantly with the progression of stage. However, in terms of VT, only stage 3 FEVR patients differed from controls and patients with stage 1 and 2. In addition, ordinal logistic regression models showed a strong correlation between peripapillary VW and FEVR severity (based on the FEVR stage). After controlling for confounding factors such as sex, age, gestational age, and birth weight, the perioptic disc VW was significantly independently correlated with the severity of FEVR, but the correlation between VT and the severity of FEVR was not significant. The results of logistic regression models also indicated that the risk of FEVR staging progression increased by 55.71% for every 1 μm increase in perioptic disc VW. Based on the above results, we can see that the increased diameter and tortuosity of the vessels around the optic disc may be one of the pathological features of FEVR.

Due to defects in retinal vascular development, patients with FEVR exhibit a high degree of venous-venous and arteriovenous shunts and a high degree of nonperfusion in peripheral capillaries [6, 9, 27, 28]. Therefore, the tortuosity and dilatation of the posterior pole vessels in patients with FEVR may be related to the presence of venous-venous and arteriovenous anastomoses in the retina. At present, there is no systematic research on this aspect, further studies are needed to determine the relationship between vascular anastomosis and vessel tortuosity and dilation in the posterior pole. In addition, defects in retinal vascular development in FEVR patients lead to increased VEGF production in the hypoxic retina, which may also contribute to the peripapillary vascular tortuosity and dilation. Although our study did not include patients with stage 4 FEVR, mechanical dragging of the macula and optic disc that is difficult to identify with the naked eye may still exist in patients with stage 3. The mechanical dragging of peripheral retinal lesions on the macula and optic disc in FEVR patients may also be one of the reasons for vascular curvature [6].

Our findings showed that FEVR patients had higher peripapillary VD compared with controls, which increased with progressing stage. Furthermore, logistic regression models demonstrated a significant correlation between peri-optic disc VD and FEVR severity. After controlling for confounders such as sex, age, gestational age at birth, and birth weight, VD around the optic disc was significantly independently associated with the severity of FEVR. The results of logistic regression models also indicated that, each 1% increase in peripapillary VD was correlated with an 88.03% increase in the risk of FEVR staging progression. Thus, we can find that increased peripapillary VD may also be another pathological feature of FEVR. In this study, we used ${6\times OD}_{r}$ as the radius to select an ROI. The VD was assessed as the ratio of the vessel area to the area of the ROI with the optic disc removed. Therefore, the VD measured in this experiment is mainly affected by three factors: the number of blood vessels, the diameter of the blood vessels and the size of the optic disc. Yuan et al. discovered that patients with asymptomatic FEVR had more blood vessels radiating from the optic disc in the posterior pole and a significantly smaller optic disc [5, 11, 12]. In addition, our experimental results showed that FEVR patients had more dilated perioptic disc vessels. In summary, FEVR has more perioptic disc vessels, greater dilated peripapillary vessels and smaller optic disc, which results in a significant increase in perioptic disc VD in FEVR patients.

In the present study, FEVR patients showed a significant increase in perioptic disc VD. In addition, Chen et al. indicated that the VD in the superficial and deep layers of the fovea decreased in patients with FEVR, and in humans, the disappearance of the superficial and deep retinal capillary systems was not observed [8–10]. However, mice in *Lrp5*^−/−^, *Ndpy*^−/−^, *Fz*d4^−/−^ and Tspan12^−/−^ all showed delayed primary retinal angiogenesis, a sparser density of blood vessels on the retinal vitreous surface, and a complete absence of a secondary and tertiary vascular system [25, 26, 29]. In summary, we can find that, in humans and mice, the same genetic mutation appears to cause diametrically opposed retinal vascular phenotype. A possible explanation for this incredible experimental result is that in the absence of canonical Wnt signaling such as Norrin or Fzd4, the retinal vascular phenotype is determined by a major defect in Wnt signaling and a compensatory response induced by hypoxia [29]. Furthermore, for the analysis of differences between human and mouse retinal vascular phenotypes, we may have been ignoring a simple but important fact: the development of retinal vessels in humans occurs in utero, whereas the development of retinal vessels in mice occurs in vitro.

In order to further explain the different retinal vascular phenotypes produced by the same gene mutation in mice and humans, based on published research results [29, 30] and our conjectures, we propose the following hypothesis: the hypoxic environment in utero has a protective effect on the development of human retinal vasculature. During human retinal vascular development, due to defective Wnt signaling, retinal vascular development is further maintained through other angiogenesis signals that are independent of the Wnt signaling pathway. With the differentiation and maturation of retinal neurons and glial cells, their metabolic demands increase, creating a radial center-to-periphery gradient of hypothesized “physiological hypoxia”. Due to the relatively hypoxic environment in utero, the gradient of “physiological hypoxia” in the retina is preserved, which maintains the growth of new blood vessels from the center to the periphery while further inducing the development of a retinal capillary layer. However, in mice, whose retinal vascular development occurs in vitro, the relatively high oxygen partial pressure in vitro disrupts the physiological hypoxia induced by the increased metabolic demand of the retina, thus interfering with the compensatory effect of other Wnt-independent angiogenesis signaling pathways in the presence of defective Wnt signaling. This in turn leads to the reduced density of blood vessels on the surface of the vitreoretinal surface in mice and the loss of the secondary and tertiary capillary layers in the retina. Further studies are needed to verify this hypothesis.

To further evaluate the possible value of peripapillary vascular parameters in monitoring changes in FEVR, we used the t-SNE algorithm to visualize FEVR severity and the vascular characteristics (VT, VW, VD) analyzed in this paper. The visualization results show that the vascular parameters have the characteristics of continuity along the direction of FEVR severity. This study used the 5-classification system proposed by Pendergast et al. to describe the staging of FEVR [13]. FEVR occurs due to a congential failure of retinal vascular development, these staging systems do not represent stages through which the disease advances [1]. However, our findings based on t-SNE visualization demonstrate a trend of continuous changes in vascular parameters as FEVR staging progresses. Therefore, monitoring the changes in perioptic disc vascular parameters may provide a new direction for monitoring the progression of FEVR. In the future, we will use the long-term follow-up of FEVR patients to clarify the value of peripapillary vascular parameters in monitoring the evolution of the disease.

Admittedly, our study has some limitations. Firstly, the sample size is relatively small, and it is necessary to expand the sample size to repeat the results of this research. In addition, neonates did not undergo FAG due to the risks of general anesthesia and allergic reaction to sodium fluorescein. So, the possibility of overlap between phase 1 and phase 2 FEVR cannot be completely ruled out. Moreover, Although the causative gene was identified by genetic testing in all enrolled FEVR patients, for the FEVR population, the causative gene could only be found in less than 50% of FEVR patients [1, 31, 32], which would result in the exclusion of FEVR patients without genetic abnormalities. Finally, the present study is retrospective and only reveals the differences in peripapillary vascular parameters between FEVR patients and normal subjects, and their relationship with the severity of disease. Further longitudinal prospective studies of vascular parameters will help to understand the pattern of changes in these indicators in the recurrence and regression of FEVR.

In conclusion, we found that the increased tortuosity, width and density of the vessels around the optic disc may be one of the pathological features of FEVR. Quantitative measurement of peripapillary vascular parameters can be used as one of the indicators to assess the severity of FEVR.

## Data Availability

The datasets generated and analysed during the current study are not publicly available due the fact that they constitute an excerpt of research in progress but are available from the corresponding author on reasonable request.

## Abbreviations

FEVR: Familial exudative vitreoretinopathy
VT: Vessel tortuosity
VW: Vessel width
VD: Vessel density
aOR: Adjusted odds ratios
OCTA: Optical coherence tomography angiography
BV: Blood vessels
OD: Optic disc
OD*r*: Radius of the optic disc
ROI: Region of interest
t-SNE: t-Distributed stochastic neighbor embedding
VEGF: Vascular endothelial growth factor.
